# Prognostic Significance of Non-Sustained Ventricular Tachycardia Depends on Its Rate and Duration

**DOI:** 10.5935/abc.20180078

**Published:** 2018-05

**Authors:** Serdar Bozyel

**Affiliations:** University of Health and Sciences, Derince Training and Research Hospital, Department of Cardiology, Kocaeli - Turkey

**Keywords:** Heart Failure / physiopathology, Tachycardia, Ventricular, Hospitalization, Defibrillators Implantables

**Dear Editor,**

We read the article “Non-Sustained Ventricular Tachycardia (NSVT) Episodes Predict Future Hospitalization in ICD Recipients with Heart Failure” written by Uçar et al.^1^ with great interest. NSVT identified on routine ICD interrogation should be considered an important clinical event as the authors state in this article. However, we would like to bring attention to some issues related to the article.

NSVT was defined as 4 or more consecutive beats with a rate > 167 beats/min and shorter than 16 beats in this study. Both detection rate and number of intervals to detect (NID) ventricular tachycardia were slightly below the conventional interval (NID = 18/24) detection of VT/VF ≥ 188 bpm that have been proven effective.^2^^,^^3^ If we also include the new long-detection programming strategies (NID = 30/40) into this subject, we can say that the authors documented the increase in hospitalization, just only with slower and shorter episodes of NSVT.

Previously published reports showed that faster and longer runs of NSVT were more predictive than slower and shorter ones for adverse events.^4^ But, since there was no data about duration and rate of the NSVT episodes in the article, we could not establish an opinion about the importance of rate and duration of NSVT for predicting future hospitalizations.

More recently, the use of long-detection interval programming has received widespread acceptance based on several large randomized trials.^3^^,^^5^ With these new programming strategy, we believe that the prognostic value of NSVT will increase even more.

## References

[1] Uçar FM, Yilmaztepe MA, Taylan G, Aktoz M (2017). Non-sustained ventricular tachycardia episodes predict future
hospitalization in ICD recipients with heart failure. Arq Bras Cardiol.

[2] Wathen MS, DeGroot PJ, Sweeney MO, Stark AJ, Otterness MF, Adkisson WO (2004). Prospective randomized multicenter trial of empirical
antitachycardia pacing versus shocks for spontaneous rapid ventricular
tachycardia in patients with implantable cardioverter defibrillators: Pain
FREER x II Trial Results. Circulation.

[3] Gasparini M, Proclemer A, Klerasy C, Kloppe A, Lunati M, Ferrer JB (2013). Effect of long-detection interval vs standard-detection interval
for implantable cardioverter-defibrillators on antitachycardia pacing and
shock delivery: the ADVANCE III randomized clinical trial. JAMA..

[4] Wang W, Lian Z, Rowin EJ, Maron BJ, Maron HS, Link MS (2017). Prognostic implications of nonsustained ventricular tachycardia
in high-risk patients with hypertrophic cardiomyopathy. Circ Arrhythm Electrophysiol..

[5] Wilkoff BL, Williamson BD, Stern RS, Moore SL, Lu F, Lee SW, PREPARE Study Investigators (2008). Strategic programming of detection and therapy parameters in
implantable cardioverter defibrillators reduces shocks in primary prevention
patients:results from the PREPARE (Primary Prevention Parameters Evaluation)
study. J Am Coll Cardiol.

