# Supplementary material for: Prognostic Significance of Non-Sustained Ventricular Tachycardia Depends on Its Rate and Duration
Source: Arq Bras Cardiol. 2018 May;110(5):495–6. doi: 10.5935/abc.20180078 (PMC5967147; doi:10.5935/abc.20180078)
Supplement: Related texts

# Reply

**Authors:** Fatih Mehmet Uçar

**Dear Editor,**

We thank the journal readers for their great interest in our original article titled “Non-Sustained Ventricular Tachycardia Episodes Predict Future Hospitalization in ICD Recipients with Heart Failure” recently published in *Arquivos Brasileiros de Cardiologia.*^1^

In our study, we programmed ICD’s zones as ventricular tachycardia VT (167-200 bpm) with discriminators and VF (> 200 bpm). *Non-sustained ventricular tachycardia*NSVT was defined in the monitored zone of ICD as 4 or more consecutive beats arising below the atrioventricular node with a rate > 167 beats/min and shorter than 16 beats. We used the number of intervals to detect (NID) ventricular tachycardia in VF zone or fast VT(FVT) zone. In our device program FVT zone was off NID was 18/24 in VF zone. If we programmed NID as 30/40, maybe we could detect more NSVTs, but in our analyses, we did not have any NSVT patients in VF (> 200 bpm) zone. All our patients were in VT (167-200 bpm) zone.

Finally, it is difficult to make a final decision according to our findings with a relatively limited study population. Hence, the new long-detection programming strategies need to be confirmed in further and larger prospective multicenter trials about the prognostic value of NSVT.

Sincerely,  Fatih Mehmet Uçar 

## References

[1] Uçar FM, Yilmaztepe MA, Taylan G, Aktoz M (2017). Non-sustained ventricular tachycardia episodes predict future
hospitalization in ICD recipients with heart failure. Arq Bras Cardiol.
